# Single progesterone receptor-positive phenotype has the similar clinicopathological features and outcome as triple-negative subtype in metastatic breast cancer

**DOI:** 10.3389/fonc.2023.1029648

**Published:** 2023-02-24

**Authors:** Yunbo Luo, Hongyu Pu, Fangwei Li, Shuangqiang Qian, Jingtai Chen, Xiaobo Zhao, Lingmi Hou

**Affiliations:** ^1^ Department of Thyroid and Breast Surgery, Affiliated Hospital of North Sichuan Medical College, Nanchong, China; ^2^ Department of Thyroid and Breast Surgery, Chongqing People’s Hospital, Chongqing, China; ^3^ Laboratory of Thyroid (Parathyroid) and Breast Disease, Affiliated Hospital of North Sichuan Medical College, Nanchong, China; ^4^ Department of Academician (expert) Workstation, Biological Targeting Laboratory of Breast Cancer, Breast and Thyroid Surgery, Affiliated Hospital of North Sichuan Medical College, Nanchong, China

**Keywords:** metastatic breast cancer, single progesterone receptor-positive, endocrine therapy, chemotherapy, outcome

## Abstract

**Objective:**

The same clinicopathological features and prognosis have been reported between single progesterone receptor-positive (sPR-positive) and triple-negative phenotype in early-stage breast cancer, but such similarity has not been studied in metastatic breast cancer (MBC). Therefore, the purpose of this study was to estimate the difference between sPR-positive phenotype and other phenotypes in MBC.

**Methods:**

Patients with HER-2-negative MBC were selected from the Surveillance, Epidemiology and End Results database. Pearson’s χ2 test was used to compare the difference of clinicopathologic factors between sPR-positive phenotype and other phenotypes. Univariate and multivariate analyses were performed to evaluate the effects of hormone receptor (HoR) phenotypes and other clinicopathologic factors on the cancer-specific survival (CSS) and overall survival (OS).

**Results:**

Overall, 10877 patients including 7060 patients (64.9%) with double HoR-positive (dHoR-positive), 1533 patients (14.1%) with single estrogen receptor-positive (sER-positive), 126 patients (1.2%) with sPR-positive and 2158 patients (19.8%) with double HoR-negative (dHoR-negative) were analyzed. The patients with sPR-positive or dHoR-negative were more likely to be younger, higher grade and tumor stage, visceral and brain metastasis than ER-positive phenotypes (P<0.001). MBC with sPR-positive had the similar CSS (HR: 1.135, 95%CI: 0.909-1.417, P=2.623) and OS (HR: 1.141, 95%CI: 0.921-1.413, P=0.229) as dHoR-negative, but worse outcome than ER-positive phenotypes. Chemotherapy significantly improved the survival for MBC, especially for sPR-positive MBC (CSS, HR: 0.39, 95%CI: 0.213-0.714, P=0.002; OS, HR: 0.366, 95%CI: 0.203-0.662, P=0.001).

**Conclusions:**

Patients with sPR-positive and triple-negative have similar biological behavior and prognosis in MBC. Chemotherapy may be a preferred recommendation for MBC with sPR-positive.

## Introduction

Breast cancer is the most common malignant tumor in women and seriously threatens their health and lives (1). Fortunately, after the finding of hormone receptors (HoR) including estrogen receptor (ER) and progesterone receptor (PR), endocrine therapy was gradually becoming the standard treatment for patients with HoR-positive breast cancer and significantly improved the survival for those patients (2). With the development of endocrine therapy, many traditional endocrine therapy regimens including tamoxifen, ovarian function suppression, aromatase inhibitor and fulvestrant have contributed greatly to the survival of patients with HoR-positive breast cancer (2–5). In addition, the combination of cyclin-dependent kinase 4/6 inhibitors and the above endocrine drugs becomes a better choice for patients with HoR-positive breast cancer, especially for metastatic breast cancer (MBC) (6).

More than 80% of breast cancers are HoR-positive (7), and the National Comprehensive Cancer Network (NCCN) guidelines recommend endocrine therapy for patients with ER-positive (ER+) and/or PR-positive (PR+). Actually, there are four HoR phenotypes including double HoR-positive phenotype (ER+/PR+, dHoR-positive), single ER-positive phenotype (ER+/PR-, sER-positive), single PR-positive phenotype (ER-/PR+, sPR-positive) and double HoR-negative (ER-/PR-, dHoR-negative). Many experts have suspected the existence of sPR-positive phenotype and thought it resulted from technical artifacts (8–10), but more and more evidence has justified the existence of this phenotype both in biology and clinic (11, 12). Many previous studies have explored the causes of sPR-positive breast cancer and demonstrated that the major mechanism is the secondary loss of ER (13–15). Furthermore, multiple studies have demonstrated that the breast cancer with sPR-positive and HER-2-negative phenotype has the same clinicopathological characteristics as triple-negative subtype and is also not sensitive to endocrine therapy (11, 16–19). However, those studies included the patients with stage I-III breast cancer but not MBC. Although the most recent study included patients with MBC, the proportion of MBC in the statistical analysis was very small (20). Therefore, we used the stage IV breast cancer with HER-2-negative at the initial diagnosis from the Surveillance, Epidemiology and End Results (SEER) database to analyze the clinicopathological difference between sPR-positive phenotype and other HoR phenotypes.

## Material and methods

### Data source and patient selection

**Figure 1:**
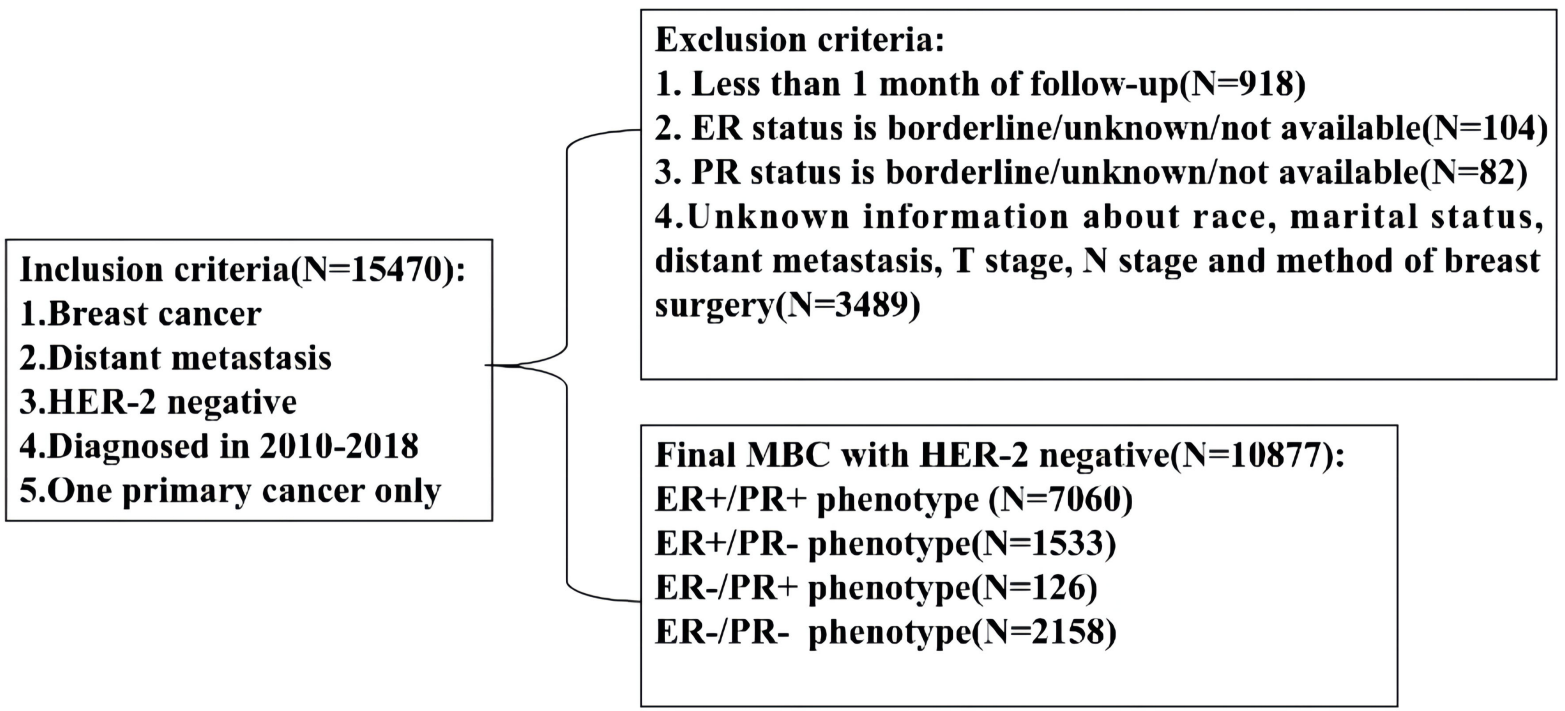
Flowchart for patient selection from the Surveillance, Epidemiology and End Results (SEER) database. MBC, metastatic breast cancer; ER, estrogen receptor; PR, progesterone receptor.

Retrospective study was performed by using the National Cancer Institute’s SEER database which covers approximately 28% of the United States population. Because the SEER database began collecting the HER-2 status and distant metastatic sites from 2010, our study employed the data of SEER database from 2010 to 2018. SEER*Stat version 8.3.8 (http://seer.cancer.gov/seerstat) was used to identify the eligible patients based on the following inclusion criteria: breast cancer, definite distant metastasis, HER-2-negative status, years of diagnosis from 2010 to 2018, one primary cancer only, available HoR status and other clinicopathological information (**Figure 1**, flowchart). Finally, 10877 patients were enrolled in our study and their information including sex, age, race, marital status, histology type, grade, tumor and lymph node stage, ER and PR status, metastatic sites, therapeutic methods and survival months were collected and analyzed. Because the personally identifiable information about patients could not be obtained from the SEER database, our study was approved to be exempt from ethical review by ethics Committee of our institution.

### Statistical analysis

The enrolled patients were divided into four cohorts including ER+/PR+, ER+/PR-, ER-/PR+ and ER-/PR- according to HoR status. Then, Pearson’s χ2 test was used to estimate the clinicopathologic difference among these four cohorts. The cancer-specific survival (CSS) and overall survival (OS) were the endpoints of our study. CSS was defined as the interval from the diagnosis of breast cancer to death caused by breast cancer or the final follow-up in censored cases, and OS was defined as the interval from diagnosis of breast cancer to death from all causes or the last follow-up in censored cases. Survival differences were assessed through Kaplan-Meier analysis, followed by a log−rank test. Then, the multivariable Cox proportional hazards model was used and hazard ratios (HR) with the corresponding 95% confidence intervals (CI) were subsequently calculated to estimate the independent prognostic factors. STATA software (Version 13; Stata Corporation) was applied for all statistical analyses. The forest plot was generated by Microsoft Office Excel (Version 2021; Microsoft Corporation). All tests were two sided and p value <0.05 were considered statistically significant.

## Results

### Patient characteristics

A total of 10877 patients were diagnosed with HER-2-negative MBC at initial presentation between 2010 and 2018 and were included in this study. Among them, 7060 patients (64.9%) were dHoR-positive, 1533 patients (14.1%) were sER-positive, 126 patients (1.2%) were sPR-positive and 2158 patients (19.8%) were dHoR-negative (**Table 1**). The patients with sPR-positive or dHoR-negative were more likely to be younger and higher percentage of black race when compared with dHoR-positive or sER-positive (P<0.001). A lower proportion of patients with sPR-positive or dHoR-negative presented invasive lobular carcinoma than patients with dHoR-positive or sER-positive (P<0.001). Furthermore, the patients with sPR-positive or dHoR-negative presented higher histological grade and tumor stage than patients with dHoR-positive or sER-positive (P<0.001). Less bone metastasis occurred to patients with sPR-positive (40.5%) or dHoR-negative (42.8%) than patients with dHoR-positive (76%) or sER-positive (68%), but more visceral and brain metastasis happened to patients with sPR-positive or dHoR-negative than patients with dHoR-positive or sER-positive (P<0.001). More patients with sPR-positive (42.9%) or dHoR-negative (40.5%) got surgery of the breast than patients with dHoR-positive (30.1%) or sER-positive (30.9%). Also, more patients with sPR-positive (71.4%) or dHoR-negative (80.6%) accepted chemotherapy than patients with dHoR-positive (52.8%) or sER-positive (58.1%).

**Table 1:** The clinicopathological features of patients with HER-2-negative MBC in different hormone receptor status.

Variables	N (%)	ER+/PR+, N (%)	ER+/PR-, N (%)	ER-/PR+, N (%)	ER-/PR-, N (%)	P value
Total	10877 (100)	7060 (64.9)	1533 (14.1)	126 (1.2)	2158 (19.8)	
Age at diagnosis						0.001
≤60	5271 (48.5)	3395 (48.1)	674 (44)	68 (54)	1134 (52.5)	
>60	5606 (51.5)	3665 (51.9)	859 (56)	58 (46)	1024 (47.5)	
Sex						0.001
Female	10726 (98.6)	6939 (98.3)	1518 (99)	125 (99.2)	2144 (99.4)	
Male	151 (1.4)	121 (1.7)	15 (1)	1 (0.8)	14 (0.6)	
Race						<0.001
White	8111 (74.6)	5442 (77.1)	1136 (74.1)	77 (61.1)	1456 (67.4)	
Black	1866 (17.1)	989 (14)	283 (18.5)	36 (28.6)	558 (25.9)	
Others	900 (8.3)	629 (8.9)	114 (7.4)	13 (10.3)	144 (6.7)	
Marital status						0.474
Married	5000 (46)	3281 (46.5)	699 (45.6)	55 (43.7)	965 (44.7)	
Unmarried	5877 (54)	3779 (53.5)	834 (54.4)	71 (56.3)	1193 (55.3)	
Histological type						<0.001
IDC	7714 (70.9)	4904 (69.5)	1018 (66.4)	96 (76.2)	1696 (78.6)	
ILC	1275 (11.7)	992 (14.1)	229 (14.9)	6 (4.8)	48 (2.2)	
IDC and ILC	494 (4.6)	378 (5.3)	66 (4.3)	2 (1.6)	48 (2.2)	
Others	1394 (12.8)	786 (11.1)	220 (14.4)	22 (17.4)	366 (17)	
Grade						<0.001
I-II	4987 (45.8)	3964 (56.1)	653 (42.6)	15 (11.9)	355 (16.5)	
III-IV	4263 (39.2)	2025 (28.7)	604 (39.4)	95 (75.4)	1539 (71.3)	
Unknown	1627 (15)	1071 (15.2)	276 (18)	16 (12.7)	264 (12.2)	
Tumor stage						<0.001
T_0-2_	5134 (47.2)	3504 (49.6)	716 (46.7)	50 (39.7)	864 (40)	
T_3-4_	5743 (52.8)	3556 (50.4)	817 (53.3)	76 (60.3)	1294 (60)	
Lymph node stage						<0.001
N_0_	3858 (35.5)	2585 (36.6)	562 (36.7)	42 (33.3)	669 (31)	
N_1-2_	5137 (47.2)	3427 (48.5)	686 (44.7)	56 (44.5)	968 (44.9)	
N_3_	1882 (17.3)	1048 (14.9)	285 (18.6)	28 (22.2)	521 (24.1)	
Bone metastasis						<0.001
No	3497 (32.2)	1697 (24)	490 (32)	75 (59.5)	1235 (57.2)	
Yes	7380 (67.8)	5363 (76)	1043 (68)	51 (40.5)	923 (42.8)	
Lung metastasis						<0.001
No	7660 (70.4)	5155 (73)	1136 (74.1)	75 (59.5)	1294 (60)	
Yes	3217 (29.6)	1905 (27)	397 (25.9)	51 (40.5)	864 (40)	
Liver metastasis						<0.001
No	8713 (80.1)	5846 (82.8)	1194 (77.9)	92 (73)	1581 (73.3)	
Yes	2164 (19.9)	1214 (17.2)	339 (22.1)	34 (27)	577 (26.7)	
Brain metastasis						<0.001
No	10236 (94.1)	6753 (95.7)	1423 (92.8)	113 (89.7)	1947 (90.2)	
Yes	641 (5.9)	307 (4.3)	110 (7.2)	13 (10.3)	211 (9.8)	
Chemotherapy						<0.001
Yes	6450 (59.3)	3730 (52.8)	891 (58.1)	90 (71.4)	1739 (80.6)	
No	4427 (40.7)	3330 (47.2)	642 (41.9)	36 (28.6)	419 (19.4)	
Radiation						0.079
Yes	3909 (35.9)	2548 (36.1)	583 (38)	40 (31.7)	738 (34.2)	
No	6968 (64.1)	4512 (63.9)	950 (62)	86 (68.3)	1420 (65.8)	
Surgery						<0.001
Yes	3525 (32.4)	2123 (30.1)	473 (30.9)	54 (42.9)	875 (40.5)	
No	7352 (67.6)	4937 (69.6)	1060 (69.1)	72 (57.1)	1283 (59.5)	

MBC, metastatic breast cancer; ER, estrogen receptor; PR, progesterone receptor; IDC, invasive ductal carcinoma; ILC, invasive lobular carcinoma.

### Univariate survival analysis

**Figure 2:**
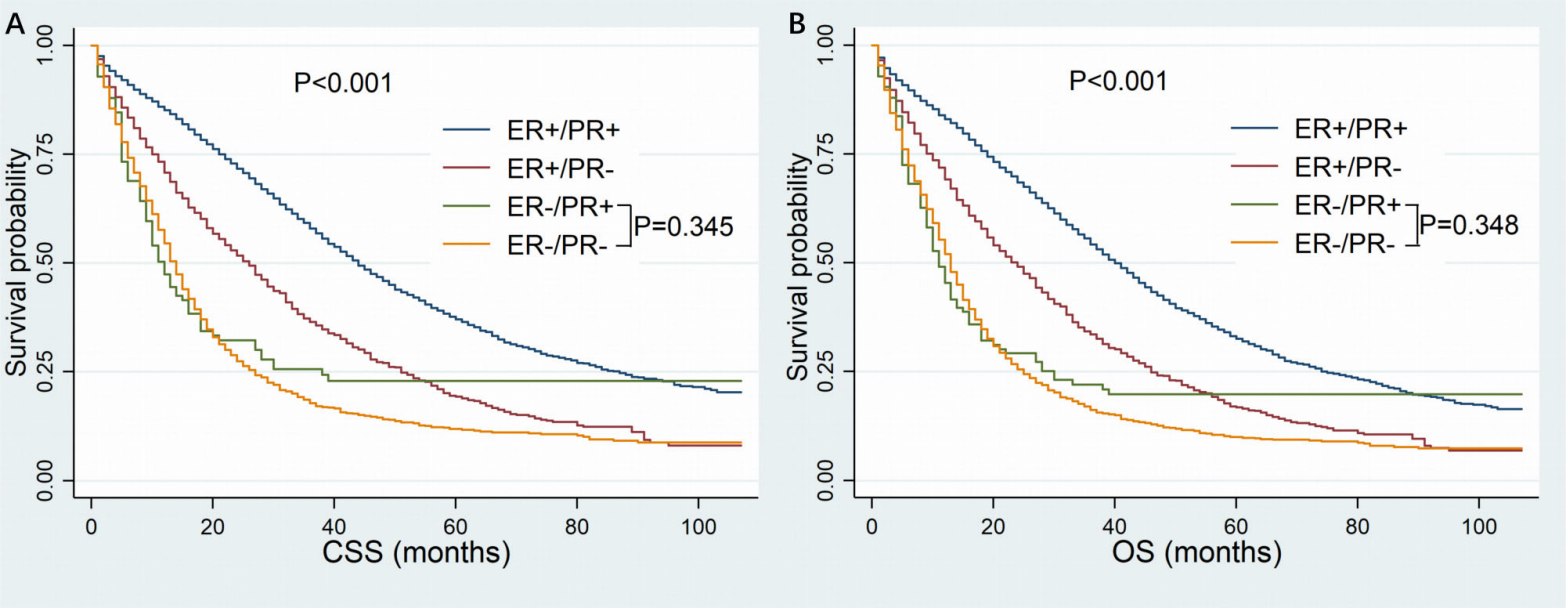
Kaplan-Meier curves of cancer-specific survival **(A)** and overall survival **(B)** based on hormone receptor status for patients with HER-2-negative metastatic breast cancer. CSS, cancer-specific survival; OS, overall survival; ER, estrogen receptor; PR, progesterone receptor.

The follow-up time ranged from 1 to 106 months, with a median of 19 months. Finally, death occurred to 6381 patients including 3633 patients with dHoR-positive, 1006 patients with sER-positive, 89 patients with sPR-positive and 1653 patients with dHoR-negative. As shown in **Figure 2**, the patients with sPR-positive had the same CSS as patients with dHoR-negative (median CSS: 12 and 14 months, respectively, P=0.345), but both had significantly worse CSS than patients with dHoR-positive (median CSS: 44 months, P<0.001). Also, the patients with sPR-positive had the same OS as patients with dHoR-negative (median OS: 11 and 13 months, respectively, P=0.348), but both had worse OS than patients with dHoR-positive (median OS: 40 months, P<0.001). In addition to HoR status, other clinicopathologic factors could also have impacts on the survival of patients with MBC. As shown in **Table 2**, worse CSS and OS were seen in those patients who were older, black race, unmarried status, higher histological grade (III-IV), higher tumor stage (T_3–4_), visceral and brain metastasis. Anti-tumor treatments including radiation, chemotherapy and especially surgery of the breast could significantly extend the survival for patients with MBC.

**Table 2:** Unadjusted CSS and OS for patients with HER-2-negative MBC.

Variables	Number (%)	Cancer-specific survival (CSS)		Over survival (OS)	
		Median CSS (months)	P value	Median OS (months)	P value
Total	10877 (100)				
Age at diagnosis					
≤60	5271 (48.5)	36	<0.001	34	<0.001
>60	5606 (51.5)	31		27	
Sex					
Female	10726 (98.6)	33	0.968	30	0.787
Male	151 (1.4)	36		29	
Race					
White	8111 (74.6)	35	<0.001	32	<0.001
Black	1866 (17.1)	23		29	
Others	900 (8.3)	38		35	
Marital status					
Married	5000 (46)	38	<0.001	36	<0.001
Unmarried	5877 (54)	29		26	
Histological type					
IDC	7714 (70.9)	33	<0.001	30	<0.001
ILC	1275 (11.7)	40		36	
IDC and ILC	494 (4.6)	41		37	
Others	1394 (12.8)	26		23	
Grade					
I-II	4987 (45.8)	46	<0.001	41	<0.001
III-IV	4263 (39.2)	23		21	
Unknown	1627 (15)	29		27	
HoR status					
ER+/PR+	7060 (64.9)	44	<0.001	40	<0.001
ER+/PR-	1533 (14.1)	26		24	
ER-/PR+	126 (1.2)	12		11	
ER-/PR-	2158 (19.8)	14		13	
Tumor stage					
T_0-2_	5134 (47.2)	40	<0.001	37	<0.001
T_3-4_	5743 (52.8)	28		26	
Lymph node stage					
N_0_	3858 (35.5)	35	0.009	31	0.056
N_1-2_	5137 (47.2)	34		30	
N_3_	1882 (17.3)	30		27	
Bone metastasis					
No	3497 (32.2)	28	0.002	25	0.001
Yes	7380 (67.8)	35		32	
Lung metastasis					
No	7660 (70.4)	37	<0.001	34	<0.001
Yes	3217 (29.6)	24		21	
Liver metastasis					
No	8713 (80.1)	38	<0.001	34	<0.001
Yes	2164 (19.9)	19		18	
Brain metastasis					
Yes	10236 (94.1)	35	<0.001	32	<0.001
No	641 (5.9)	12		12	
Radiation					
Yes	3909 (35.9)	36	<0.001	33	<0.001
No	6968 (64.1)	32		29	
Surgery					
Yes	3525 (32.4)	46	<0.001	42	<0.001
No	7352 (67.6)	29		26	
Chemotherapy					
Yes	6450 (59.3)	34	<0.001	32	<0.001
No	4427 (40.7)	33		28	

CSS, cancer-specific survival; OS, overall survival; MBC, metastatic breast cancer; HoR, hormone receptor; ER, estrogen receptor; PR, progesterone receptor; IDC, invasive ductal carcinoma; ILC, invasive lobular carcinoma.

### Multivariate survival analysis

**Figure 3:**
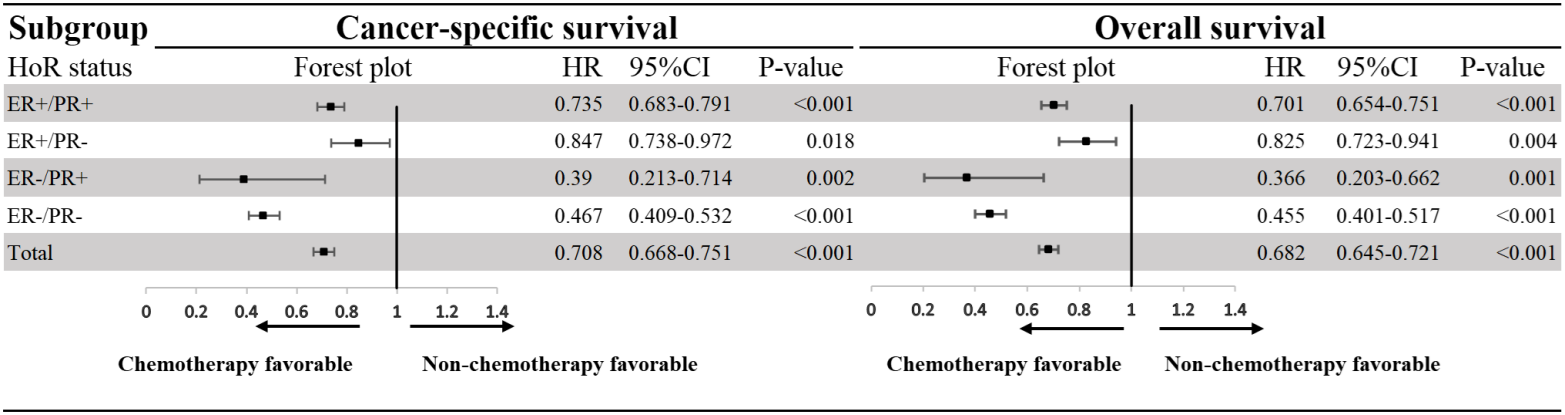
Effect of chemotherapy on patients with HER-2-negative metastatic breast cancer according to hormone receptor status. HoR, hormone receptor; ER, estrogen receptor; PR, progesterone receptor; HR, hazard rations; CI, confidence intervals.

When multivariate survival analysis was performed (**Table 3**), better outcomes were seen in patients with ER-positive. Especially in patients with dHoR-positive, multivariate survival analysis shown significant better CSS (HR: 0.366, 95%CI: 0.293-0.458, P<0.001) and OS (HR: 0.382, 95%CI: 0.309-0.474, P<0.001) compared with patients of sPR-positive. Also, the patients with sER-positive exhibited better CSS (HR:0.624, 95%CI: 0.497-0.784, P<0.001) and OS (HR:0.625, 95%CI: 0.501-0.778, P<0.001) than patients with sPR-positive. However, patients with dHoR-negative had the same CSS (HR: 1.135, 95%CI: 0.909-1.417, P=0.263) and OS (HR: 1.141, 95%CI: 0.921-1.413, P=0.229) compared with patients of sPR-positive. Then, the older age, black race, unmarried status, invasive lobular carcinoma, higher histological grade (III–IV), higher tumor stage (T_3-4_), visceral (lung and liver) and brain metastasis were independent risk factors for OS and CSS. Surgery of the breast and chemotherapy obviously increased the survival for MBC. Furthermore, the subgroup survival analysis shown that chemotherapy significantly improved the CSS (HR: 0.39, 95%CI: 0.213-0.714, P=0.002) and OS (HR: 0.366, 95%CI: 0.203-0.662, P=0.001) for patients with sPR-positive (**Figure 3**).

**Table 3:** Multivariable Cox regression for CSS and OS among patients with HER-2-negative MBC.

Variables	Cancer-specific survival (CSS)			Overall survival (OS)		
	HR	95%CI	P value	HR	95%CI	P value
Age at diagnosis						
≤60	Ref			Ref		
>60	1.195	1.132-1.262	<0.001	1.247	1.184-1.314	<0.001
Sex						
Female	Ref			Ref		
Male	1.229	0.982-1.539	0.072	1.230	0.995-1.520	0.055
Race						
White	Ref			Ref		
Black	1.198	1.119-1.282	<0.001	1.221	1.144-1.302	<0.001
Others	0.902	0.815-0.998	0.045	0.923	0.839-1.016	0.102
Marital status						
Married	Ref			Ref		
Unmarried	1.221	1.158-1.289	<0.001	1.254	1.192-1.320	<0.001
Histological type						
IDC	Ref			Ref		
ILC	1.155	1.056-1.263	0.002	1.134	1.042-1.234	0.004
IDC and ILC	1.151	1.013-1.309	0.031	1.144	1.013-1.292	0.03
Others	1.073	0.990-1.162	0.085	1.087	1.008-1.172	0.031
Grade						
I-II	Ref			Ref		
III-IV	1.477	1.386-1.575	<0.001	1.428	1.343-1.517	<0.001
Unknown	1.174	1.082-1.273	<0.001	1.135	1.051-1.227	0.001
HoR status						
ER-/PR+	Ref			Ref		
ER+/PR+	0.366	0.293-0.458	<0.001	0.382	0.309-0.474	<0.001
ER+/PR-	0.624	0.497-0.784	<0.001	0.625	0.501-0.778	<0.001
ER-/PR-	1.135	0.909-1.417	0.263	1.141	0.921-1.413	0.229
Tumor stage						
T0-2	Ref			Ref		
T3-4	1.243	1.177-1.312	<0.001	1.240	1.178-1.306	<0.001
Lymph node stage						
N0	Ref			Ref		
N1-2	0.996	0.940-1.056	0.906	0.989	0.935-1.045	0.69
N3	1.061	0.983-1.144	0.13	1.047	0.973-1.126	0.219
Bone metastasis						
No	Ref			Ref		
Yes	1.274	1.200-1.354	<0.001	1.241	1.172-1.315	<0.001
Lung metastasis						
No	Ref			Ref		
Yes	1.245	1.175-1.319	<0.001	1.227	1.161-1.296	<0.001
Liver metastasis						
No	Ref			Ref		
Yes	1.754	1.649-1.866	<0.001	1.710	1.611-1.815	<0.001
Brain metastasis						
No	Ref			Ref		
Yes	1.811	1.639-2.001	<0.001	1.788	1.624-1.968	<0.001
Surgery						
Yes	Ref			Ref		
No	1.698	1.596-1.808	<0.001	1.693	1.595-1.796	<0.001
Radiation						
Yes	Ref			Ref		
No	1.021	0.964-1.082	0.484	1.037	0.982-1.096	0.194
Chemotherapy						
Yes	Ref			Ref		
No	1.412	1.332-1.497	<0.001	1.467	1.388-1.550	<0.001

CSS, cancer-specific survival, OS, overall survival; HR, hazard ratios; CI, confidence intervals; MBC, metastatic breast cancer; HoR, hormone receptor; ER, estrogen receptor; PR, progesterone receptor; IDC, invasive ductal carcinoma; ILC, invasive lobular carcinoma.

## Discussion

Detection of hormone receptors can provide prognostic information for breast cancer patients (21, 22), and also endocrine therapy can significantly improve the survival for patients with HoR-positive (2). Thus, the accuracy of hormone receptors testing becomes very critical for breast cancer patients. In 2010, the American Society of Clinical Oncology/College of American Pathologists (ASCO/CAP) published the guideline for ER/PR immunohistochemical (IHC) detection, which clearly proposed that the expression level of ER/PR should be detected in all newly diagnosed breast cancer patients, and emphasized the basic operating procedures, quality control and result interpretation criteria of ER/PR detection (23). This guideline defined 1% as the threshold for positive ER/PR expression in IHC, and recommended that the percentage of positive cells and the intensity of positive staining should be noted in the report. Double HoR-positive phenotype occurs in the majority of patients with breast cancer and has better outcome than single HoR-positive phenotypes including sER-positive phenotype and sPR-positive phenotype (11, 16–18, 24, 25). There was a controversy that whether sPR-positive phenotype is an error or entity. Some experts attributed the sPR-positive phenotype to artifacts arising from the preparation or assay of the sample, such as inadequate tissue fixation or technique failure of the IHC assay (10, 26). However, some studies confirmed its existence through IHC (11, 12, 27). Besides, subsequent researches justified its presence through analyzing PAM50 expression signature and mRNA level of ESR1, which also revealed that 53-65% of patients with sPR-positive phenotype were basal-like and didn’t respond well to endocrine therapy (28, 29). Recent studies shown that the sPR-positive phenotype has the same characteristics as dHoR-negative phenotype and may not well respond to endocrine therapy (11, 16–18, 20). But those studies didn’t include or included a small percentage of patients with MBC which clearly differs from early-stage breast cancer. Therefore, we estimated the eligible patients from SEER database to figure out if the biological behavior of MBC with sPR-positive is the same as early-stage breast cancer reported by previous researches.

Consistent with previous studies (11, 12, 16, 17, 26), the patients with sPR-positive accounted for 1.2% of the whole cohort in our study. Also, our study exhibited the same clinicopathological features between sPR-positive phenotype and dHoR-negative phenotype, such as, younger age, less proportion of invasive lobular carcinoma, higher histologic grade, later tumor stage and more lymph nodes involved. What has not been reported is the difference of metastatic sites between breast cancer with sPR-positive phenotype and other phenotypes. Our study shown the metastatic tendency of sPR-positive phenotype kept with dHoR-negative phenotype and it was more likely to be visceral and brain metastasis for sPR-positive phenotype compared with ER-positive phenotypes. This finding further sheds light on the similar aggressive biological behavior between sPR-positive phenotype and dHoR-negative phenotype in MBC.

Compared with sPR-positive phenotype, the patients with dHoR-positive or sER-positive phenotype significantly exhibited better outcomes. While, the same prognosis between sPR-positive phenotype and dHoR-negative phenotype was seen in our study. The difference of prognosis among these four cohorts keeps with previous studies (11, 16, 17). Multiple studies (30, 31) have reported that surgery of the breast can improve the survival for stage IV breast cancer, which was also proved in our study. Then, chemotherapy as the main treatment to delay the progression of MBC can also significantly improve the survival for MBC, especially for such patients with sPR-positive phenotype as shown in the forest plot. This interesting finding was also reported in a previous study which used propensity score matching cohorts to show the significant benefit from chemotherapy for sPR-positive phenotype (17). The remarkable effect of chemotherapy on MBC with sPR-positive phenotype may be due to the insensitivity of this phenotype to endocrine therapy. Unfortunately, the information about endocrine therapy can’t be acquired from SEER database. Although the explicit endocrine therapy information can’t be obtained, most of the patients with ER-positive and/or PR-positive would have received appropriate endocrine therapy for the wide use of NCCN guidelines. Bardou, et al (18) performed a retrospective study including patients from two large breast cancer databases to evaluate whether progesterone receptor status provided prediction of benefit from endocrine treatment. One of the cohorts including 1688 patients of endocrine therapy shown that sPR-positive phenotype had the same outcome compared with dHoR-negative phenotype, and another cohort containing 10444 patients of endocrine therapy also demonstrated that result. In addition, a large meta-analysis including 20 trails shown that 1236 patients with sPR-positive phenotype didn’t benefit from adjuvant tamoxifen therapy (Rate ration=0.9, 95CI%: 0.73-1.12, P=0.35) (19). Actually, previous studies have revealed that only 20-30% of patients with sPR-positive breast cancer are luminal-like and the majority are basal-like (28, 29, 32), which explained why patients with sPR-positive didn’t significantly benefit from endocrine therapy. The right treatments are crucial for MBC because the noneffective therapeutic regimens may lead to tumor progression and finally worsen the outcome. Therefore, the MBC with sPR-positive should be dealt with seriously and chemotherapy can be the most crucial treatment for such group for its outstanding effect on improving survival as shown above. Meanwhile, the gene expression measurement should be performed to find the minority patients of sPR-positive phenotype belonging to luminal-like and endocrine therapy should also be used to palliate the progression of MBC.

Several limitations of this study must be elucidated. Firstly, some bias is inevitable due to retrospective nature of this study. Secondly, endocrine therapy information is not available from the SEER database and we can’t directly analysis the effect of endocrine therapy on MBC with sPR-positive. Finally, the number of patients with sPR-positive is not very large, so the conclusion of our study must be further justified by larger population. However, our study is the first one that used the MBC with HER-2-negative to analysis the difference between sPR-positive phenotype and other phenotypes in clinicopathological features and survival. And it further confirms the similar biological behavior between sPR-positive phenotype and triple-negative phenotype in MBC, which can guide the clinicians to make better treatment strategies when facing with this rare phenotype.

## Conclusions

MBC with sPR-positive and HER-2-negative has the similar biological behavior to triple-negative MBC, such as younger age, higher histological grade, larger tumor burden and predisposition to visceral and brain metastasis. The MBC with sPR-positive and HER-2-negative has the similar prognosis to MBC of triple-negative but worse prognosis than ER-positive phenotype. Chemotherapy may be a preferred recommendation for patients with sPR-positive phenotype because it significantly improves their survival.

## Data availability statement 

The datasets presented in this study can be found in online repositories. The names of the repository/repositories and accession number(s) can be found below: the Surveillance, Epidemiology, and End Results (SEER) database (https://seer.cancer.gov).

## Ethics statement

Ethical review and approval was not required for the study on human participants in accordance with the local legislation and institutional requirements. Written informed consent for participation was not required for this study in accordance with the national legislation and the institutional requirements.

## Author contributions

YL, XZ, and LH conceived and designed this study. YL, HP, FL, and JC collected and analyzed the data. YL, SQ, and HP organized the manuscript. YL, XZ, and LH reviewed the paper and revised the manuscript. All authors contributed to the article and approved the submitted version.
